# Manual and automatic assignment of two different Aβ40 amyloid fibril polymorphs using MAS solid-state NMR spectroscopy

**DOI:** 10.1007/s12104-024-10189-z

**Published:** 2024-08-09

**Authors:** Natalia Rodina, Riddhiman Sarkar, Dimitrios Tsakalos, Saba Suladze, Zheng Niu, Bernd Reif

**Affiliations:** 1 Department of Bioscience, TUM School of Natural Sciences, Technical University of Munich, Munich, Germany; 2 Institute of Structural Biology, Helmholtz Zentrum Munich or German Research Center for Environmental Health, Munich, Germany; 3 School of Pharmacy, Henan University, Kaifeng, China

**Keywords:** Solid-state NMR, Carbon-detection, Amyloid fibrils, Abeta peptide, Assignment, Automated assignment

## Abstract

Amyloid fibrils from Alzheimer’s amyloid-beta peptides (Aβ) are found to be polymorphic. So far, 14 Aβ40 fibril structures have been determined. The mechanism of why one particular protein sequence adopts so many different three-dimensional structures is yet not understood. In this work, we describe the assignment of the NMR chemical shifts of two Alzheimer’s disease fibril polymorphs, P1 and P2, which are formed by the amyloid-beta peptide Aβ40. The assignment is based on ^13^C-detected 3D NCACX and NCOCX experiments MAS solid-state NMR experiments. The fibril samples are prepared using an extensive seeding protocol in the absence and presence of the small heat shock protein αB-crystallin. In addition to manual assignments, we obtain chemical shift assignments using the automation software ARTINA. We present an analysis of the secondary chemical shifts and a discussion on the differences between the manual and automated assignment strategies.

**Supplementary Information:**

The online version contains supplementary material available at 10.1007/s12104-024-10189-z.

## Biological context

Deposition and accumulation of ordered protein aggregates - amyloid fibrils - in tissues and organs accompanies various serious neurodegenerative diseases such as Alzheimer’s and Parkinson’s disease, type II diabetes, cataracts, systemic AA amyloidosis, etc. (Dobson 1999). The development of techniques for solving amyloid fibril structures, such as solid-state nuclear magnetic resonance spectroscopy (NMR) and cryo-electron microscopy (cryo-EM), revealed the polymorphic nature of amyloid fibrils (Tycko 2014). Despite the general similarity that was initially discovered with the help of X-ray crystallography, fibrils from various proteins and, moreover, from the same protein display a great variety of structures (Ke et al. 2020). It is one of the most challenging and intriguing tasks in the field of amyloid fibril structural biology to find a link between fibril structure and its pathological role in the corresponding disease.

As of May 2024, 1070 amyloid structures have been solved using cryo-EM and deposited in the PDB databank (https://www.rcsb.org/stats). At the same time, only 48 amyloid structures were solved using solid-state NMR. On the other hand, the number of chemical shifts obtained from solid-state NMR amyloid samples in the BMRB database is steadily increasing (https://bmrb.io). In total, 292 chemical shift data sets were deposited that were obtained from solid-state NMR experiments. 65 of these were determined from amyloid fibril samples. Although cryo-EM has proven to be a more efficient method for the initial structure determination of fibrils, it fails to provide information on dynamic regions of the fibrils (Reif et al. 2021). Such dynamic parts, called ‘fuzzy-coat’, have recently been identified as key regions for fibril formation and their interactions with the environment and possible inhibitors (Khare et al. 2023; Ulamec et al. 2020). As of today, NMR spectroscopy is the only experimental method that can shed light on the structure and features of the unstructured parts of the fibrils. Therefore, developing solid-state NMR methodology to study amyloid fibrils remains of great importance.

A time-limiting step in NMR studies, especially in the solid-state, is the assignment of the NMR chemical shifts. In the past years, several tools for automated analysis of NMR data, including peak picking, structure determination, and resonance assignment, were developed (Bartels et al. 1997; Garrett et al. 2011; Guerry and Herrmann 2011; Güntert 2009; Hu et al. 2011; Klukowski et al. 2018; Li et al. 2021; Schmidt and Güntert 2012; Würz and Güntert 2017; Zimmerman et al. 1997). In early 2022, a cloud computing service, NMRtist (https://nmrtist.org), was released for fully automated analysis of protein NMR spectra using deep-learning approaches. A machine learning-based method was introduced as part of the platform ARTINA (Klukowski et al. 2022). ARTINA takes the protein sequence and spectra as input for solution-state NMR and provides signal positions, resonance assignments, and structures as output. In the case of solid-state NMR, manually picked peaks must be provided to perform NMR resonance assignment. Since the training sets for solid-state NMR are not sufficiently big, the reliability of those automated assignments can still be improved.

In the current work, we focus on assigning and characterizing two very similar yet different polymorphs of amyloid fibrils formed by Alzheimer’s disease β-amyloid peptide 1–40 (Aβ40). Aggregation of Aβ peptides in the brain tissue is a biomarker of Alzheimer’s disease (Tanzi and Bertram 2005). The rather small 40-residue peptide represents one of the most studied amyloid models. So far, over 14 different Aβ40 fibril structures have been determined (Bertini et al. 2011; Kollmer et al. 2019; Lu et al. 2013; Paravastu et al. 2008; Pfeiffer et al. 2024). However, not all possible morphologies seem to be uncovered yet. We have shown recently that the small heat shock protein αB-crystallin inhibits the propagation of the seed structure (polymorph P1) in Aβ40 fibrils and leads to the formation of a new polymorph (P2) with an unstructured N-terminus (Rodina et al. 2024). The two polymorphs in our study do not match the already published structures. Therefore, we performed chemical shift assignments for both polymorphs using classical carbon-detected solid-state NMR experiments and compared our manual assignments with an automated assignment performed by ARTINA(Klukowski et al. 2022).

## Methods and experiments

### Protein expression and purification

A pET28(+) vector carrying the DNA sequence coding for the Aβ40 peptide as an insert was used to transfer competent BL21(DE3) cells. To grow *E.Coli* cultures isotopically enriched M9 medium (^15^NH_4_Cl (0.5 gL^− 1^),^13^C glucose (2 gL^− 1^)) or ^15^N^13^C labeled Silantes medium were used. Overexpression was induced from addition of 1 mM isopropyl-b-d-thio-galactopyranoside (IPTG) when transformed cells reached an optical density of ∼0.6–0.7 at 600 nm (OD_600_). Cells were harvested after overexpression for 4 h at 37℃ by centrifugation (130 rpm). The produced Aβ40 peptide is accumulated in inclusion bodies (IBs). Cell lysis from 1 L culture was performed by resuspending the cell pellet in 50 ml 20 mM Tris·HCl buffer (pH 8.0) containing 20 mg*mL^− 1^ DNAse I and 2 tablets of complete protease inhibitor (Roche). The suspension was sonicated for 5 min on ice ((30% amplitude, 1 s pulse on, 1 s pulse off). The supernatants were disposed, and the pellets containing the IBs further washed in the next steps.

IBs containing Aβ40 were washed twice by incubation followed by sonification and centrifugation for 30 min (24,000 rcf, 4 °C). In the first wash step, 25 ml 20 mM Tris buffer (pH 8.0) containing 0.4% Triton-100 and 1 tablet of complete protease inhibitor was used. In the second wash step, IBs were resuspended in 25 ml 20 mM Tris buffer (pH 8.0). Each wash step was followed by incubation at RT for 10 min with gentle shaking and further sonification on ice (3 min, 30%, 1 s pulse on, 1 s pulse off). The IBs were solubilized by resuspending the pellet in 20 mM Tris (pH 8.0) buffer containing 6 M GdnHCl and, followed by 10 min incubation on ice followed by sonification for 3 min (30% amplitude, 1 s pulse on, 1 s pulse off). The dissolved IBs were centrifuged for 30 min (24,000 rcf, 4 °C) using a fiberlite F21-8 × 50y fixed-angle rotor (Thermo-Fisher). The supernatant was subsequently filtered through a 0.22 μm MWCO membrane. The filtered supernatant was loaded onto a reverse-phase chromatography SOURCE30 RPC column that was equilibrated using 80% buffer A (10 mM NaN_3_) and 20% buffer B (80% acetonitrile, 0.3% TFA). A gradient from 20 to 60% of buffer B was applied using a Dionex UltiMate 3000 HPLC system (Thermo Scientific). The absorbance at 200 nm was measured to detect Aβ40 peptide in the collected fractions. The concentration of the eluted peptide in each fraction was estimated using a NanoDrop 2000 spectrophotometer (Thermo Scientific). Mass spectrometry and SDS-Tris gel electrophoresis were used to verify the purity of the peptide. The fractions of the eluted peptide were lyophilized and stored at -80 °C. The recombinant Aβ peptide contains an additional N-terminal methionine that was shown previously to not alter the biochemical and biophysical properties of the peptide (Walsh, Thulin et al. 2009).

Recombinant wild-type αB-crystallin (αBC) was kindly provided by the group of Prof. Johannes Buchner, TU München. The sHSP was expressed and purified as described previously (Mainz et al. 2009; Peschek et al. 2009).

### Preparation of Aβ40 monomeric stock solutions

Lyophilized recombinant ^13^C,^15^N-labeled Aβ40 peptide was freshly dissolved in 10 mM NaOH to yield a final concentration of approximately 200 µM before each experiment. The solution was sonified twice for 5 min in a water bath; in between the sonication cycles, as well as before and after, the peptide solution was kept on ice. To remove pre-aggregated protein, the solution was centrifuged for 20 min at 21,000 rcf at 4 °C. In addition, the supernatant was filtered through a filter with a 0.2 μm MWCO membrane. This was followed by another round of centrifugation for 30 min at 21,000 rcf, at 4 °C. The concentration of the peptide stock solution was determined by recording an absorption spectrum using a high-precision cell 10 mm quartz cuvette (Hellma Analytics) in a V-750 spectrophotometer (Jasco, Japan), assuming the extinction coefficient *ε*_*280*_ = 1,490 M^− 1^‧cm^− 1^ that was calculated using the ProtParam online tool (Gasteiger et al. 2005).

### Seed preparation

Preformed fibrils (seeds) were used as a template to obtain a homogeneous fibril preparation. In order to obtain the initial polymorph 1 (P1), we employed a protocol that starts from a 50 µM monomeric Aβ40 solution in 50 mM phosphate buffer (pH 7.4, supplemented with 50 mM NaCl, 0.1% NaN_3_) and involves 12 generations of seeding at 37 °C, as described previously (Lopez del Amo et al. 2012). Fibrils were grown over a period of 10 days (Aβ40 monomer concentration: 50 µM) under constant shaking in the presence of 5% (w/w) seeds and 50 mM phosphate buffer (pH 7.4, supplemented with 50 mM NaCl, 0.1% NaN_3_) at 37 °C. The initial polymorph 2 (P2) fibrils were obtained by incubating a 50 µM monomeric Aβ40 solution with 5% (w/w) P1 seeds that were premixed right before the start of the experiment using a 5 µM solution of the small heat shock protein alpha-B-crystallin (αBC). The solution was incubated for 2 weeks under constant agitation in a 50 mM phosphate buffer (pH 7.4, supplemented with 50 mM NaCl, 0.1% NaN_3_) at 37 °C. αBC was washed away by sedimenting the fibrils at 4 °C and 21.000 rcf. The supernatant was carefully removed, and the pellet was resuspended in the same amount of fresh buffer. To ensure that all αBC is washed away, this procedure was repeated 5 times. Finally, the fibrils were dialyzed against a fresh buffer at 4 °C overnight. To prepare seeds, fibrils were sonicated in a glass vial in a water bath sonicator for 5 (first generation) or 10 (second generation) minutes in a water bath for both P1 and P2. Sonication was performed directly before using the seeds. The preparation of P2 seeds is described in more detail in Rodina et al. (Rodina et al. 2024).

### Preparation of the fibril sample for solid-state NMR

Fibril growth was carried out in glass vials under constant shaking at 160 rpm (Innova 40, New Brunswick Scientific) at 37 °C. To yield the final fibril sample two rounds of seeding were performed. The first generation used 2 mg of labeled peptide for each preparation. The stock solution was diluted with 50 mM phosphate buffer (pH 7.4, supplemented with 50 mM NaCl, 0.1% NaN_3_) to a final concentration of 50 µM (0.224 mg/ml). After the pH shift, 5% (w/w) seeds were added. The sample was incubated for 3 days. The fibrils obtained this way were used as seeds and mixed with a fresh batch of Aβ40 diluted with 50 mM phosphate buffer (pH 7.4, supplemented with 50 mM NaCl, 0.1% NaN_3_) to a final concentration of 50 µM. The total amount of ^13^C,^15^N labeled Aβ40 in the second generation was 10 mg. The second generation of fibrils was grown for a period of 10 days. The grown fibrils of the second generation were visualized with transmission electron microscopy (TEM). Mature fibrils from each preparation were collected by centrifugation at 21,000 rcf (4 °C), and supernatants were carefully removed. 1.9 mm ZrO_2_ (Bruker Corporation, USA) magic angle spinning (MAS) rotors were packed by sedimenting ~ 8 mg of material using the spiNpack 1.9 mm rotor packing tool (Giotto Biotech) and an ultracentrifuge (Optima L100 XP, Beckman Coulter, USA) at 28,000 rcf at 12 °C using a SW32Ti swinging bucket rotor. The packed rotors were kept at 4 °C.

### Transmission electron microscopy (TEM)

Continuous carbon-coated copper grids (Ted Pella, Inc, USA) were glow-charged for 30 s under reduced pressure. 5 µl of a 50 µM sample was incubated on the grid for 90 s. Phosphate salts were removed by washing with 20 µl ddH_2_O. For staining, 5 µl of a 2% uranyl acetate solution was applied for 45 s. A JEOL 1400 plus microscope (JEOL, Japan) was used to take micrographs at various magnifications. ImageJ (National Institute of Health, USA) was employed to process, analyze, and scale the images.

### Solid-state NMR spectroscopy

2D and 3D solid-state NMR spectra of uniformly labeled ^13^C,^15^N-labeled Aβ40 fibril samples were recorded using a Bruker Avance III 750 MHz spectrometer equipped with a triple-resonance (^1^H,^13^C,^15^N) 1.9 mm MAS probe. The MAS rotation frequency was adjusted to 16.65 kHz and sample temperature was maintained at 10 °C using cooling gas with a flow rate of 550 L/h. During acquisition, high-power proton decoupling (ωRF/2π = 100 kHz) was applied using SPINAL-64. Cross-polarization was employed for the ^1^-^13^ C magnetization transfer. ^13^C,^13^C transfers were achieved via PDSD or DARR using a mixing time of 30 or 50 ms (Takegoshi et al. 2003). Conventional 3D NCA/NCO, NCACX and NCOCX experiments were recorded for further chemical shift assignment (McDermott et al. 2000; Pauli et al. 2001). For each polymorph, two sets of experiments (without non-uniform sampling (NUS) and with NUS) were recorded (Hyberts et al. 2010). Optimal Control was employed for pulse shape optimization in selective coherence transfer between ^15^N and ^13^C (aliphatic or CO) (Baldus et al. 1998; Tošner et al. 2018). Spectra were processed in TopSpin 3.5 and 4.0.7 (Bruker Corporation, USA). 3D spectra recorded with NUS were reconstructed using NMRPipe (Hyberts et al. 2010). ^13^C and ^15^N chemical shift referencing was achieved using an external standard, N-formyl-[U-^13^C,^15^N]-L-Met-L-Leu-L-Phe (MLF) purchased from Cambridge Isotope Laboratories (Andover, MA). The exact chemical shifts of the ^13^C and ^15^N are provided in Hong et al. (Hong and Griffin 1998) and Jaroniec et al. (Jaroniec et al. 2001). Chemical shift assignments were carried out using CCPN 2.4. and CCPN 3.1.1 (Collaborative Computational Project for NMR) (Skinner et al. 2016; Stevens et al. 2011; Vranken et al. 2005). To yield assignments, the strategy described in Pradhan et al. 2021 was employed (Pradhan et al. 2021). For automated chemical shift assignment by ARTINA, manually picked peak lists for PDSD, NCA, NCACX and NCOCX spectra were provided (Klukowski et al. 2022). To predict random coil chemical shifts, the tab2bmrb tool was used which was developed by Dr. Jaravine (Dept. of Structural Biology, BIOZENTRUM, Basel University), and was provided by BMRBJ (https://bmrb.io/tab2bmrb/). The predicted random coil chemical shifts were validated using an online tool provided by the SBiNLab at the University of Copenhagen (https://www1.bio.ku.dk/english/research/bms/sbinlab/randomchemicalshifts1/) (Kjaergaard and Poulsen 2011; Schwarzinger et al. 2001). The values of the predicted random coil chemical shifts are provided in the **SI Table 3**. The secondary chemical shifts were corrected with respect to the random coil shifts and calculated as follows:

$$\eqalign{& [{C_{\alpha \>}}\left( {observed} \right) - \>{C_{\alpha \>}}\left( {random\>coil} \right)] \cr & \quad - \>[{C_{\beta \>}}\left( {observed} \right) - \>{C_{\beta \>}}\left( {random\>coil} \right)] \cr}$$

The correlation factor between secondary chemical shifts was calculated using the correl(x, y) function

$$\:correl\left(x,y\right)=\frac{\sum\:(x-\stackrel{-}{x})(y-\stackrel{-}{y})}{\sqrt{\sum\:{(x-\stackrel{-}{x})}^{2}\sum\:{(x-\stackrel{-}{x})}^{2}}}$$,

where $\:\stackrel{-}{x}$, $\:\stackrel{-}{y}$ represent the average values for x and y. Secondary structures were predicted from assigned chemical shifts using TALOS + server (Shen et al. 2009). Origin-2023 was used for plotting.

## Results

### Reproducibility of the sample preparation

**Fig. 1:**
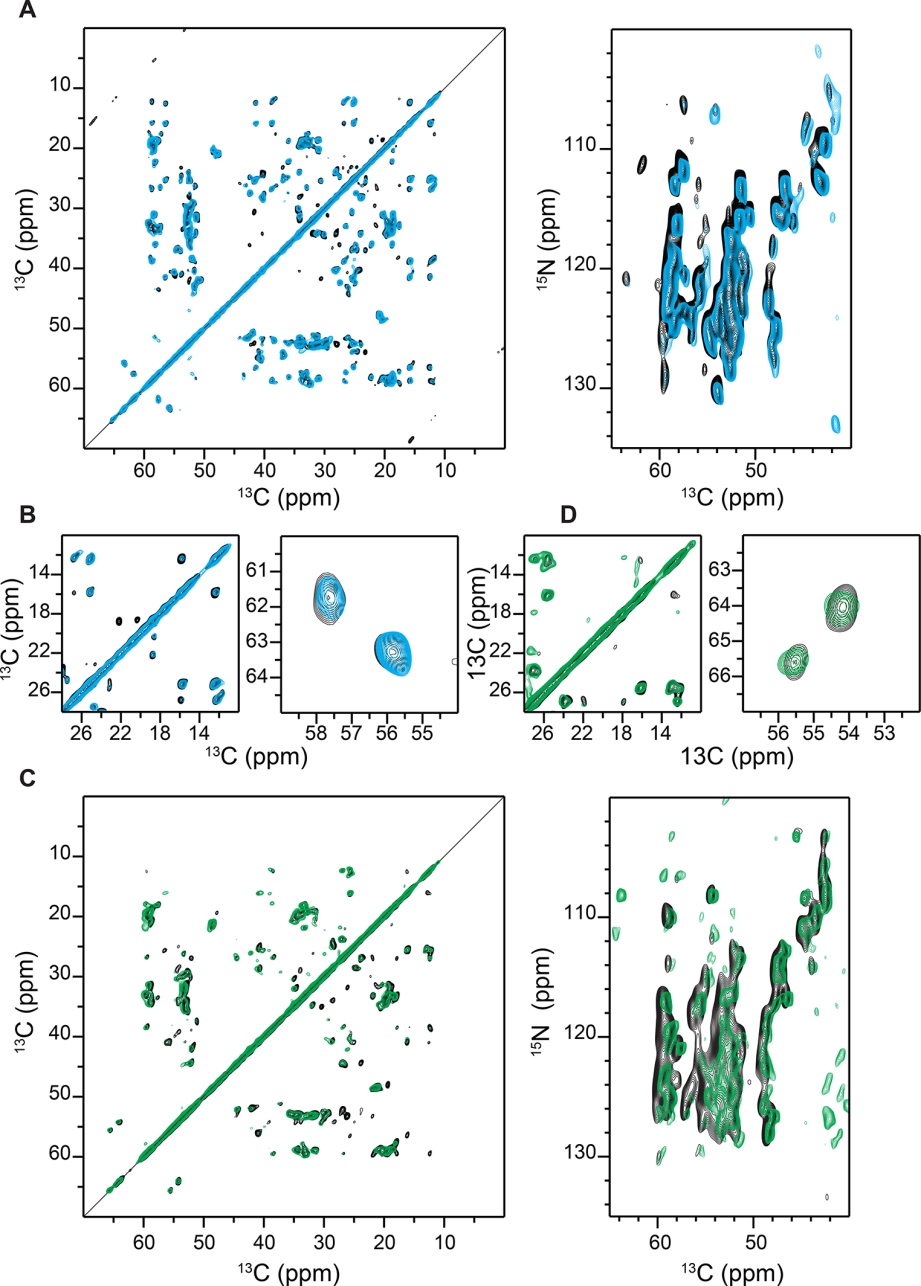
Reproducibility of the Aβ40 fibril polymorph 1 (P1) and polymorph 2 (P2) preparations. (**A**) Superposition of 2D PDSD ^13^C,^13^C (left) and 2D ^15^N,^13^C correlation spectra (right) obtained for independent P1 seeded Aβ40 fibril preparations. For clarity, we show 2 preparations out of 3. The 2 preparations are represented in black and cyan. (**B**) Ile (left) and Ser (right) regions of the 2D PDSD ^13^C,^13^C correlation spectra from Fig. 1A. (**C**) Superposition of 2D PDSD ^13^C,^13^C (left) and 2D ^15^N,^13^C correlation spectra (right) obtained for independent P2 seeded Aβ40 fibril preparations. For clarity, we show 2 preparations out of 3. The 2 preparations are represented in black and green. (**D**) Ile (left) and Ser (right) regions of the 2D PDSD ^13^C,^13^C correlation spectra from Fig. 1C

**Fig. 2:**
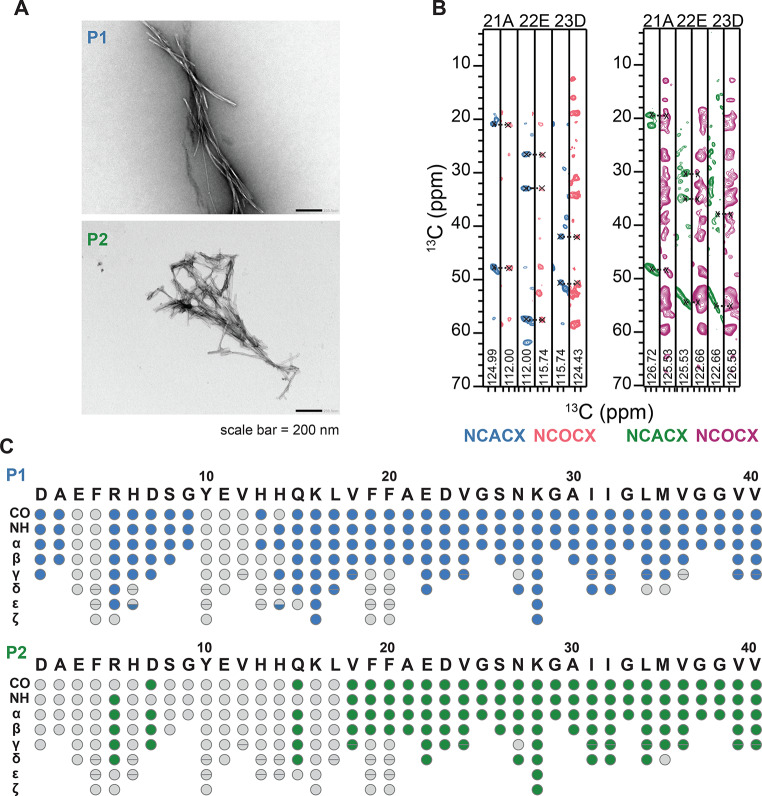
Solid-state NMR resonance assignments for P1 and P2 fibrils. (**A**) TEM images for P1 (top) and P2 (bottom) fibrils employed for solid-state NMR experiments. (**B**) 2D strip plots extracted from 3D NCACX and 3D NCOCX spectra focusing on residues 21A-23D for P1 (left) and P2 (right) fibrils. (**C**) Assigned atoms in the protein sequence for P1 (blue; top) and P2 (green; bottom) Grey-colored atoms could not be assigned

In the first step, we wanted to test whether subsequent fibril preparations using the P1 and P2 seeds yield reproducible homogeneous solid-state NMR spectra. For this purpose, three Aβ40 fibril samples were prepared for each type of seed, using 5% (w/w) P1 and P2, respectively. The preparations resulted in a very similar spectral pattern in 2D ^13^C-^13^C PDSD and 2D NCA experiments, suggesting that the structure of the fibrils is reproduced from one preparation to another (Fig. 1). Although the fibrils show a high degree of structural homogeneity, P1 and P2 appear to be different (Fig. 2, A). Inspection of the serine spectral region reveals that the P1 and P2 adopt a distinct polymorphic structure (Fig. 1, B, D). Aβ40 contains only two serine residues at positions 8 and 26 in its sequence. The appearance of exactly 2 cross peaks suggests that each preparation is homogeneous. The difference in the serine chemical shifts, however, indicates that the structures of the two polymorphs are distinct.

### Manual chemical shift assignments obtained from carbon-detected experiments

**Fig. 3:**
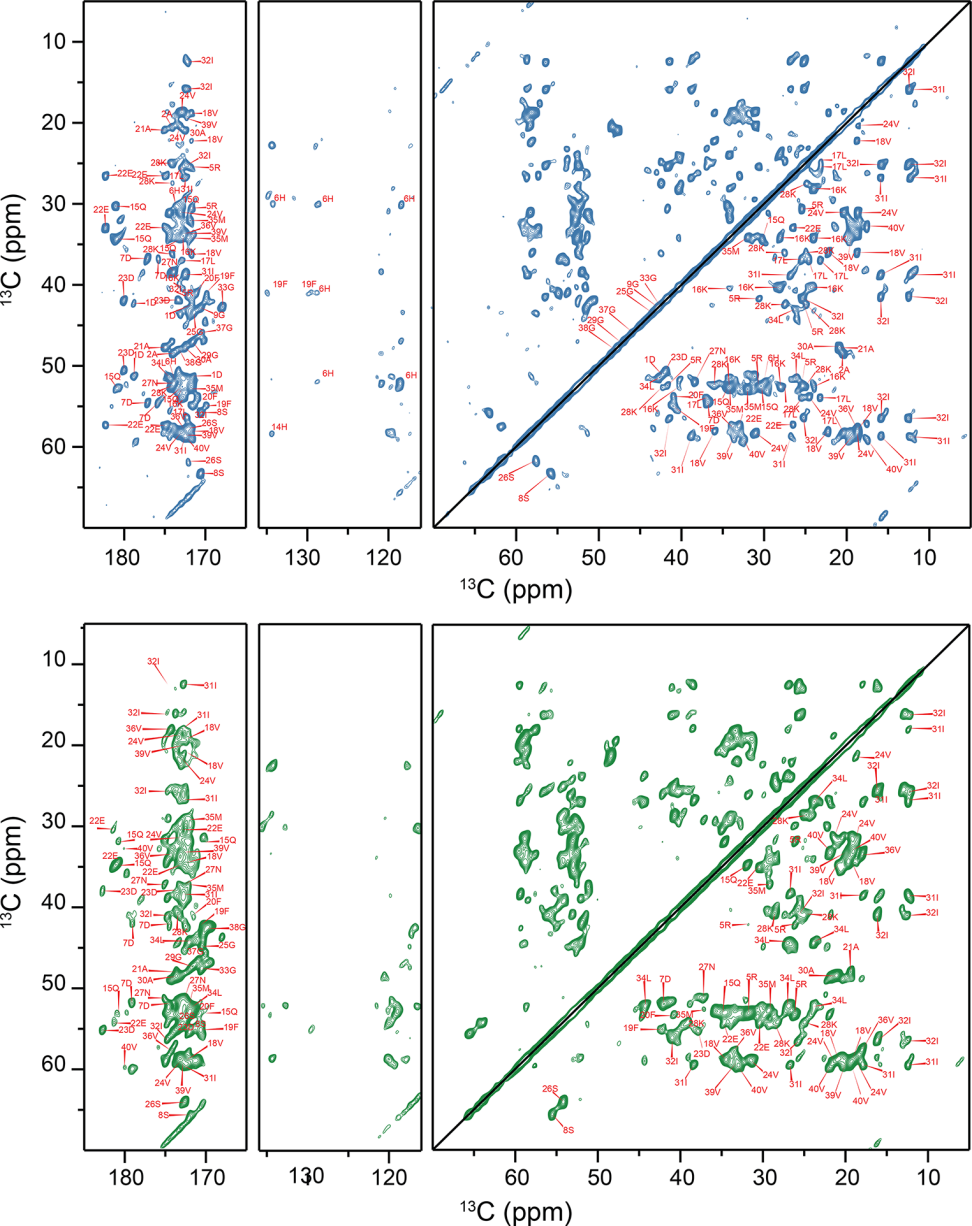
Chemical shift assignments for P1 (top) and P2 (bottom) fibrils. Carbonyl, aromatic and aliphatic regions of the 2D ^13^C,^13^C PDSD correlation spectra are shown

To identify the fibril core and flexible regions, we recorded carbon-detected 3D MAS solid-state NMR experiments using uniformly labeled (^13^C,^15^N) samples packed into 1.9 mm rotors for both P1 and P2 fibril preparations. The sequential assignments were obtained using standard 3D NCACX and 3D NCOCX experiments (McDermott et al. 2000) employing the strategy described by Pradhan et al. (Pradhan et al. 2021). Examples of 2D strips extracted from those experiments are represented in Fig. 2, B. The manual assignment procedure was repeated 3 times for each polymorph. The software packages CcpN v.2.4.2, v.2.5.2, and v.3.0.1 were employed for the manual assignment.(Skinner et al. 2016; Stevens et al. 2011; Vranken et al. 2005) To avoid false positives in peak picking, the appearance of cross-peaks with a low signal-to-noise ratio was confirmed by inspection of a second 3D dataset that was recorded independently. In total, 35 out of 40 residues for P1 and 27 out of 40 spin systems for P2 have been unambiguously sequentially assigned. The completeness of backbone assignment thus amounts to 85% and 65% for P1 and P2, respectively. 58% and 45% of all side chain heavy atoms were assigned for P1 and P2, respectively. Figure 3 shows the carbonyl, aromatic and aliphatic regions of the 2D ^13^C,^13^C PDSD spectra for P1 and P2 with assignments. Surprisingly, we were able to partially assign the first 10 amino acids in P1 fibrils, which were not visible in most preparations (Fig. 2, C) (Ghosh et al. 2021; Paravastu et al. 2008; Pfeiffer et al. 2024). We believe that the non-structured N-terminal residues in P2 fibrils form the so-called “fuzzy-coat”, a flexible region that is important for various interactions of amyloid fibrils with their environment (Ulamec et al. 2020).

### Automated chemical shift assignments from carbon-detected experiments with ARTINA

Manual chemical shift assignment is most time-limiting in the analysis of bio-NMR data. Software for automated assignment is being developed to simplify this task and to make NMR experiments more accessible. We tested one of the available artificial intelligence-based programs, ARTINA, and compared the resulting automated assignment with our manual assignments. Due to the lower sensitivity of solid-state NMR spectra and the smaller amount of available training datasets, automated peak picking has yet to be implemented for carbon-detected solid-state NMR. We, therefore, employed manually picked peak lists that were obtained in due course of the manual assignment procedure using 2D ^13^C-^13^C PDSD, 2D NCA, 3D NCACX, and 3D NCACX experiments. As it can be seen in Table 1, we identified more cross-peaks than expected for the NCACX and NCA experiments for P1. This is due to the fact that peak picking was performed manually, and in case of uncertainty, for example, overlapping peaks, all potential cross-peaks were picked.

**Fig. 4:**
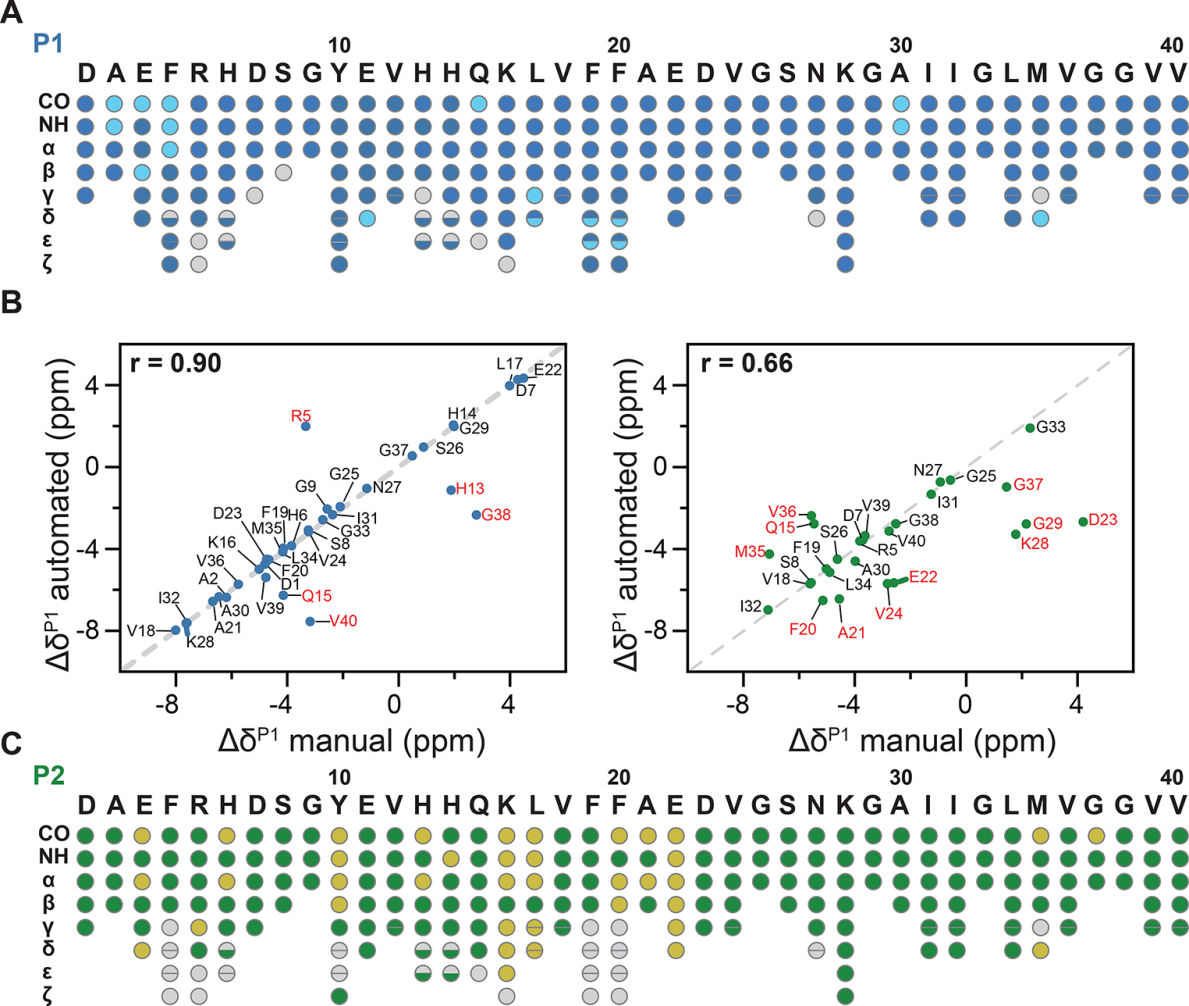
Automated resonance assignment using the software ARTINA, employing ^13^C-detected 3D experiments with manually picked peaks. **A**. Colored circles illustrate assigned atoms for P1 fibrils. Blue circles represent atoms assigned confidently by the Flya algorithm, whereas cyan circles represent uncertain assignments. **B**. Residue-specific secondary chemical shift correlation plot for P1 (left) and P2 (right). The x- and y- axis depict the experimental secondary chemical shifts for manual and automated assignments, respectively. The secondary chemical shifts are highly correlated for P1 (*r* = 0.90), suggesting that the manual and the automated assignments yield identical results. The differences for P2 are more severe (*r* = 0.66.) The secondary chemical shifts are calculated as the difference between the experimentally observed chemical shifts and the random coil chemical shift values. **C**. Colored circles illustrate assigned atoms in the protein for P2 fibrils. Green circles represent atoms assigned confidently by the Flya algorithm, whereas yellow circles represent uncertain assignments

ARTINA could assign 100% of the backbone for both P1 and P2. However, the assignment for several peaks was ambiguous (Fig. 4A, C). For P1 89.9% of the side chains were assigned, while for P2 only for 78% of all side chain atoms solutions were found. Table 1 shows the number and percentage of automatically assigned cross-peaks for each preparation using ARTINA.

**Table 1:** Completeness of automated assignment using ARTINA for P1 and P2, employing ^13^C-detected solid-state NMR experiments

		P1				P2			
experiment	expected cross-peaks	observed cross-peaks	cross peaks assigned by ARTINA	% assigned with respect to experimental cross peaks	% assigned with respect to expected peaks	observed cross-peaks	cross-peaks assigned by ARTINA	% assigned with respect to experimental cross-peaks	% assigned with respect to expected peaks
NCACX	171	185	130	70.27	76.02	149	117	78.52	68.42
NCOCX	151	129	105	81.40	69.54	115	77	66.96	50.99
PDSD	1799	487	444	91.17	24.68	513	454	88.50	25.24
NCA	41	46	29	63.04	70,73	111	29	26.13	70.73
**all**	**2162**	**847**	**708**	**83.59**	**32.75**	**888**	**677**	**76.24**	**31.31**

Interestingly, we do not obtain confident assignments for the N-terminal residues 2 A, 3E, and 4 F, as well as for 30 A in the case of P1. In manual assignments, 3E and 4 F could not be identified at all, while 2 A was clearly visible. For P1, the resonances of the residues 10Y-14 H were assigned manually and are believed to be part of a dynamic loop structure in the fibril. By contrast, ARTINA provides a confident resonance assignment for these amino acids. Overall, the automated and manually performed assignments for P1 are very similar and show a high correlation factor (*r* = 0.9) (Fig. 4B, **left**). For P2, the completeness of the assignment was on the order of 45% and 78% for the side-chain atoms in manual and automated assignments, respectively. The assignment by ARTINA differs from the manual one not only in its completeness but also in the absolute values of the chemical shifts for the core part of the sequence (Fig. 4B, **right**). With a calculated correlation factor of 0.66 the two assignments for P2 can be considered to be rather different. The accuracy of the manual assignment is supported by the fact that the procedure was performed independently three times by different researchers with the same outcome. Therefore, a more careful inspection is necessary to understand the origin of the observed discrepancies. It should be noted that in both cases, the provided peak lists play a critical role. The 3D experiments of both polymorphs were recorded under exactly the same conditions using an equal amount of labeled material in the rotor. Even though P1 and P2 fibril samples were recorded under comparable conditions, the sensitivity for the experiments recorded for P2 was significantly lower. We speculate that a lower spectral quality for P2 might be due to the increased global dynamics of this polymorph. A lower quality of the 3D spectra results in fewer picked cross peaks (Table 1), leading to a less complete manual assignment. The reduced signal-to-noise ratio might thus explain the differences between the manual and automated P2 assignments.

### Comparison of the secondary structure for P1 and P2

**Fig. 5:**
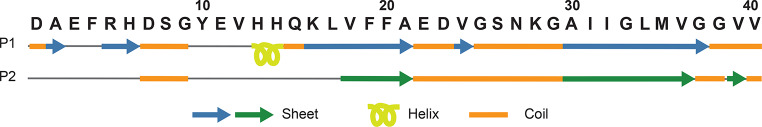
Secondary structure prediction by TALOS + from manually assigned NMR chemical shifts for polymorphs P1 and P2

Subsequently, we determined the secondary structure of both polymorphs using the chemical shift information obtained from manual assignments. In order to predict secondary structure from chemical shifts, the TALOS + server (Shen et al. 2009) was employed (Fig. 5). For P1, three β-sheets were predicted (2–6; 16–24 and 30–39). The β-sheet are separated by two major loop regions (7–15 and 25 − 20). Surprisingly, the two histidine residues at positions 13 and 14 in P1 are predicted to have a high helical propensity. However, due to the absence of chemical shift information on the preceding amino acids, formation of helical structure cannot be unambiguously confirmed. For P2, only spin systems from V18 onward could be reliably assigned in the manual assignment. Thus, no structural information on the N-terminal region of the peptide could be obtained. The two major β-sheets (18–21 and 30–39) are interrupted by a long loop involving residues 21–29. Similar to P1, the secondary chemical shifts suggest a coil involving residues 6–9.

In a recent manuscript, we used the manually obtained chemical shift assignment for a more detailed structural comparison of the two polymorphs (Rodina et al. 2024). Although the core structure of P1 and P2 has similarities, the correlation plots indicate that several residues are off the diagonal (D7, S8, A21, E22, D23, K28, G33, M35 and G38). We speculate that these residues determine the fibril topology and polymorphism. In P1, two salt-bridges (5R-7D and 6 H-22E) stabilize the N-terminus, while P2 contains one C-terminal salt bridge involving residues K28-V40. Moreover, we conducted INEPT experiments to show that there are no flexible residues (no peaks detected) for P1 fibrils, while several cross-peaks are observed for P2, indicating a flexible N-terminus.

### Structural comparison of P1 and P2 with available Ab40 structures

**Fig. 6:**
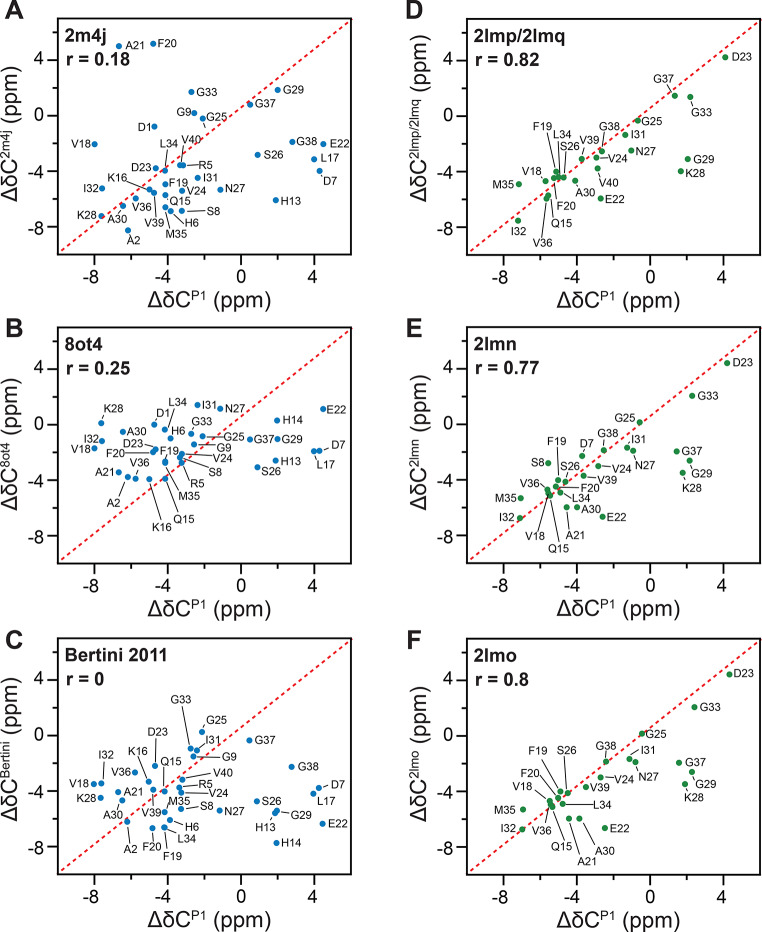
Residue-specific secondary chemical shift correlation plots. The x- and y- axes depict the experimental secondary chemical shifts for P1 (**A**-**C**) and P2 (**D**-**F**) and the published secondary chemical shifts, respectively. The corresponding correlation coefficients are represented as *r*-values. The secondary chemical shifts are calculated as the differences between the experimentally observed chemical shift and the random coil chemical shift value

Aβ40 fibrils are one of the most studied amyloid models, and many different structures have been reported in the past 20 years. From 11 Aβ40 fibril structures listed in the AmyloidAtlas (Sawaya et al. 2021), only 3 contain a structured N-terminus: 2m4j, 8ot4 and 6shs (Kollmer et al. 2019; Lu et al. 2013; Pfeiffer et al. 2024). Bertini and coworkers have reported a further fibril structure in which the N-terminus was observable and adopted a β-sheet conformation, which was detached from the core of the fibril structure (Bertini et al. 2011). Various other polymorphs with different symmetry, salt-bridge topology and morphology were identified (Cerofolini et al. 2020; Ghosh et al. 2021; Paravastu et al. 2008; Pfeiffer et al. 2024). We, therefore, compared our experimental chemical shifts for P1 with the three different topologies that contain a structured N-terminus (2m4j, 8ot4 and Bertini et al. 2011) (Table 2). For P2, we have chosen the four topologies with either a 2-fold or a 3-fold symmetry for a more detailed analysis (2lmn, 2lmo, 2lmp and 2lmq) (Table 2). Chemical shifts were obtained from the BMRB for the NMR structures. The respective BMRB IDs are shown in Table 2. The 8ot4 model by Pfeiffer et al. was obtained from cryo-EM data. We employed the shift2X tool for this conformer to predict NMR resonance frequencies (Han et al. 2011). Correlation coefficients (r) were calculated for Cα, Cβ, secondary chemical shifts (Δδ = Cα-Cβ) and CΟ (Table 2). We find high r-values for the PDBs 2lmn, 2lmo and 2lmp/2lmq structures that either adopt 2- or 3-fold symmetry. The average correlation coefficient for the morphology with 3-fold symmetry (0.79) is slightly higher than that for the 2-fold symmetric polymorph (0.71 and 0.73). The correlation plots for secondary chemical shifts are represented in Fig. 6. Unfortunately, none of the published structures with a structured N-terminus yields satisfying r-values when compared to our P1 NMR chemical shifts. We believe that P1 and P2 share a similar fibril core structure and differ mainly in the flexibility of the N-terminus and, as a consequence, in the residues that are affected by the N-terminus. We are currently working towards a structural characterization of both P1 and P2 fibrils to better understand conformational differences between P1 and P2.

**Table 2:** Comparison of P1 and P2 fibrils with selected available Aβ40 fibril structure models

	PDB ID	Ref.	Method (BMRB ID)	Fold	Region	*N*-term	K28-D23	K28-V40	*r*(Cα)	*r*(Cβ)	*r*(Δδ)	*r*(CO)	< *r*>
**P1**	2m4j	Lu et al. (2013)	NMR (19009)	3	1–40	yes	yes	no	0.22	0.06	0.18	**0.03**	0.12
	8ot4	Pfeiffer al. (2024)	cryoEM	2	1–37	yes	yes	no	0.41	0.00	0.25	0.09	0.19
		Bertini et al. (2011)	NMR (-)	2	2–40	yes	yes	no	-0.1	0.0	0.0	0.4	0.06
**P2**	**2lmn**	Paravastu et al. (2008)	NMR (18127)	2	9–40	no	yes	yes	0.76	0.68	0.77	0.61	0.71
	2lmo		NMR (18128)	2	9–40	no	yes	yes	0.77	0.73	0.80	0.64	0.73
	2lmp		NMR (18129)	3	9–40	no	yes	yes	0.83	0.64	0.82	0.86	0.79
	2lmq			3	9–40	no	yes	yes					0.79

## Conclusion

In this work, we have presented the chemical shift assignments for two Aβ40 fibril polymorphs. For P2, we obtain a topology that appears to be similar to the 3-fold symmetric structure previously determined by Paravastu et al. (PDB 2lmp, 2lmq) that exhibits a flexible N-terminus (Paravastu et al. 2008). By contrast, we could assign the N-terminal amino acids for P1. No similarities between P1 and other available Aβ40 fibril structures could be identified. To confirm our manual assignments, we tested the ARTINA software using chemical shift information from PDSD, NCACX and NCOCX experiments. We find that the reliability of automated assignments highly depends on the experimental spectra quality and that careful validation of the automated assignment is required.

## Electronic supplementary material

Below is the link to the electronic supplementary material.

Supplementary Material 1: Chemical shifts for polymorph P1 and polymorph P2 Aβ40 amyloid fibrils obtained from automated chemical shift assignment from carbon-detected MAS solid-state NMR experiments using ARTINA

## Data Availability

The manually obtained NMR chemical shift assignments for polymorph P1 and P2 are deposited in the BMRB under access codes 52337 and 52338, respectively. NMR chemical shifts assignment for polymorph P1 and P2 performed by the automated software ARTINA are available in Supplementary information Tables 1 and 2, respectively.
