# Supplementary material for: Manual and automatic assignment of two different Aβ40 amyloid fibril polymorphs using MAS solid-state NMR spectroscopy
Source: Biomol NMR Assign. 2024 Aug 9;18(2):201–12. doi: 10.1007/s12104-024-10189-z (PMC11511749; doi:10.1007/s12104-024-10189-z)
Supplement: Supplementary file 1 — Supplementary Material 1: Chemical shifts for polymorph P1 and polymorph P2 Aβ40 amyloid fibrils obtained from automated chemical shift assignment from carbon-detected MAS solid-state NMR experiments using ARTINA [file 12104_2024_10189_MOESM1_ESM.pdf]

Supporting Information for the manuscript

Manual and automatic assignment of two different Aβ40 amyloid fibril polymorphs using MAS solid-state NMR spectroscopy

Natalia Rodina[1,2]. Riddhiman Sarkar[1,2]. Dimitrios Tsakalos[1]. Saba Suladze[1]. Zheng Niu[4]. and Bernd Reif[1,2]

[1]Department of Bioscience. TUM School of Natural Sciences. Technical University of Munich. Munich. Germany
[2]Institute of Structural Biology. Helmholtz Zentrum Munich or German Research Center for Environmental Health. Munich. Germany
[4]School of Pharmacy. Henan University. Kaifeng. China

For submission to: Biomolecular NMR Assignments

To whom correspondence should be addressed: reif@tum.de

SI Table 1. Chemical shifts in ppm obtained for P1 with automated assignment by ARTINA

	RESIDUE	N	Nδ	Nε	C	Cα	Cβ	Cγ	Cδ	Cε	Cζ
**1**	**Asp**	123.36	-	-	171.54	51.28	42.34	171.55	-	-	-
**2**	**Ala**	122.56	-	-	174.04	48.34	20.59	-	-	-	-
**3**	**Glu**	120.64	-	-	171.72	52.72	32.01	34.21	171.75	-	-
**4**	**Phe**	124.06	-	-	171.74	54.69	41.61	134.96	128.81	129.66	129.66
**5**	**Arg**	128.12	-	-	171.73	52.43	25.39	25.41	41.67	-	-
**6**	**His**	126.07	-	-	173.86	52.15	30.11	128.72	118.47	134.26	-
**7**	**Asp**	126.21	-	-	175.81	54.56	36.81	175.81	-	-	-
**8**	**Ser**	121.18	-	-	170.52	55.8	63.29	-	-	-	-
**9**	**Gly**	112.7	-	-	171.55	43.26	-	-	-	-	-
**10**	**Tyr**	128.05	-	-	171.64	52.32	41.64	120.92	128.79	118.54	169.68
**11**	**Glu**	126.03	-	-	173.46	52.55	30.22	34.34	180.95	-	-
**12**	**Val**	123.52	-	-	172.68	58.25	33.88	18.77. 18.75	-	-	-
**13**	**His**	124.48	-	-	172.96	58.31	34.15	134.41	119.86	134.41	-
**14**	**His**	124.34	-	-	173.01	58.31	31.01	134.41	119.86	134.41	-
**15**	**Gln**	113.59	-	-	173.02	51.37	31	34.22	174.42	-	-
**16**	**Lys**	123.37	-	-	172.9	52.62	34.18	24	28.07	40.33	-
**17**	**Leu**	124.39	-	-	172.78	53.88	36.92	25.29	24.57. 23.20	-	-
**18**	**Val**	116.14	-	-	171.74	58.09	36.06	22.25. 18.77	-	-	-
**19**	**Phe**	125.28	-	-	169.86	54.88	40.98	134.96	129.59	129.66	128.77
**20**	**Phe**	130.38	-	-	171.13	53.82	40.99	134.96	134.96	129.31	128.77
**21**	**Ala**	124.83	-	-	174.91	47.7	20.89	-	-	-	-
**22**	**Glu**	111.92	-	-	174.87	57.33	26.53	32.93	182.27	-	-
**23**	**Asp**	115.68	-	-	173.15	50.54	41.88	180.01	-	-	-
**24**	**Val**	124.35	-	-	173.08	58.25	31	20.38. 18.73	-	-	-
**25**	**Gly**	112.58	-	-	171.49	43.41	-	-	-	-	-
**26**	**Ser**	111.33	-	-	172.01	57.57	61.76	-	-	-	-
**27**	**Asn**	117.75	-	-	173.97	52.18	38.43	173.9	-	-	-
**28**	**Lys**	117.73	-	-	174.03	52.44	36.08	24.87	27.48	42.41	-
**29**	**Gly**	115.74	-	-	171.57	47.09	-	-	-	-	-
**30**	**Ala**	115.33	-	-	172.48	47.7	20.87	-	-	-	-
**31**	**Ile**	120.52	-	-	172.41	58.69	38.74	15.88. 26.77	12.33	-	-
**32**	**Ile**	123.84	-	-	172.28	56.44	41.5	15.86. 25.24	12.47	-	-
**33**	**Gly**	109.5	-	-	167.83	42.67	-	-	-	-	-
**34**	**Leu**	125.91	-	-	171.73	51.48	42.81	26.25	26.28. 26.23	-	-
**35**	**Met**	120.62	-	-	171.74	52.75	34.09	31.99	-	17.61	-
**36**	**Val**	124.03	-	-	172.75	57.78	33.75	18.78. 18.78	-	-	-
**37**	Gly	116.07	-	-	170.51	45.9	-	-	-	-	-
**38**	Gly	112.75	-	-	170.54	42.82	-	-	-	-	-
**39**	Val	116.07	-	-	172.72	57.97	33.76	18.77. 18.77	-	-	-
**40**	Val	116.09	-	-	171.69	58.09	36.05	18.77. 22.24	-	-	-

SI Table 2. Chemical shifts in ppm obtained for P2 with automated assignment by ARTINA.

	RESIDUE	N	Nδ	Nε	C	Cα	Cβ	Cγ	Cδ	Cε	Cζ
1	Asp	122.33	-	-	173.72	55.24	37.93	182.75	-	-	-
2	Ala	119.08	-	-	173.53	48.42	21.16	-	-	-	-
3	Glu	120.43	-	-	173.27	52.11	30.25	34.61	172.03	-	-
4	Phe	125.47	-	-	173.65	56.67	40.19	-	-	-	-
5	Arg	123.45	-	91.83	172.62	53.18	31.75	26.17	40.69	-	-
6	His	124.22	-	-	172.96	57.82	33.29	116.45	116.45	-	-
7	Asp	126.86	-	-	174.54	51.98	42.07	179.15	-	-	-
8	Ser	117.24	-	-	172.09	55.48	65.53	-	-	-	-
9	Gly	110.18	-	-	170.34	43.32	-	-	-	-	-
10	Tyr	128.31	-	-	172.88	59.38	38.41	116.45	-	-	168.11
11	Glu	124.32	-	-	172	52.74	29.26	37.16	180.97	-	-
12	Val	121.03	-	-	174.36	57.62	33.41	17.97. 17.97	-	-	-
13	His	113.93	-	-	172.98	54.24	30.27	132.8	116.43	135.65	-
14	His	125.28	-	-	172.88	54.24	30.32	132.8	116.43	135.65	-
15	Gln	125.33	-	110.61	173.11	54.23	30.37	34.8	181.39	-	-
16	Lys	125.27	-	-	173.09	54.13	34.66	24.98	28.21	39.27	-
17	Leu	126.97	-	-	173.57	53.14	44.78	29.98	26.17. 22.08	-	-
18	Val	119.5	-	-	173.21	58.79	34.5	20.95. 19.24	-	-	-
19	Phe	126.95	-	-	170.76	55.09	42.14	-	-	-	-
20	Phe	128.23	-	-	170.65	53.22	42.16	-	-	-	-
21	Ala	127.9	-	-	173.33	48.2	21.27	-	-	-	-
22	Glu	120.59	-	-	172.99	54.22	33.42	34.58	174.45	-	-
23	Asp	128.01	-	-	173.11	51.41	40.9	173	-	-	-
24	Val	117.22	-	-	175.03	59.1	34.38	20.32. 19.44	-	-	-
25	Gly	110.76	-	-	170.52	44.71	-	-	-	-	-
26	Ser	108.26	-	-	172.79	54.25	63.92	-	-	-	-
27	Asn	113.79	115.12	-	175.11	51.27	37.2	175.15	-	-	-
28	Lys	115.12	-	-	173.33	54.67	33.96	24.54	28.38	40.34	-
29	Gly	107.69	-	-	169.92	42.33	-	-	-	-	-
30	Ala	126.52	-	-	173.58	47.96	19.38	-	-	-	-
31	Ile	123.35	-	-	172.78	59.4	38.45	17.93. 26.84	12.46	-	-
32	Ile	124.69	-	-	174.7	56.52	40.95	25.63. 16.15	12.55	-	-
33	Gly	114.11	-	-	171.82	47.15	-	-	-	-	-
34	Leu	122.98	-	-	174.15	52.04	44.37	26.94	23.80. 23.21	-	-
35	Met	119.35	-	-	172.1	52.91	34.54	31.72	-	18.99	-
36	Val	125.4	-	-	173.22	58.87	31.49	18.91	-	-	-
37	Gly	111	-	-	172.06	44.38	-	-	-	-	-
38	Gly	106.07	-	-	169.92	42.39	-	-	-	-	-
39	Val	124.01	-	-	173.09	59.4	33.15	19.40. 19.31	-	-	-
40	Val	125.95	-	-	173.16	59.57	33.11	19.12. 21.83	-	-	-

SI Table 3. Random coil chemical shifts predicted for Aβ40 peptide

Predicted random coil chemical shifts		by tab2bmrb tool https://bmrb.io/tab2bmrb/				by the SBiNLab online tool https://www1.bio.ku.dk/english/research/bms/sbinlab/randomchemicalshifts1/			
Residue		Cα	Cβ	C	N	Cα	Cβ	C	N
**1**	**Asp**	54.45	40.995	176.166	121.86	54.464	41.028	176.166	121.86
**2**	**Ala**	52.955	18.833	177.889	124.587	52.944	18.857	177.889	124.587
**3**	**Glu**	56.86	30.183	176.427	119.045	56.865	30.206	176.427	119.045
**4**	**Phe**	57.54	39.52	175.471	120.931	57.516	39.532	175.471	120.931
**5**	**Arg**	55.912	30.857	175.674	122.849	55.906	30.875	175.674	122.849
**6**	**His**	56.64	30.745	175.128	121.289	56.679	30.823	175.128	121.289
**7**	**Asp**	54.556	41.075	176.328	121.994	54.57	41.108	176.328	121.994
**8**	**Ser**	59.027	63.427	175.269	116.257	59.018	63.449	175.269	116.257
**9**	**Gly**	45.315	**.***	173.242	112.29	45.332	**.***	173.242	112.29
**10**	**Tyr**	57.865	38.705	175.709	118.727	57.84	38.719	175.709	118.727
**11**	**Glu**	56.262	30.279	175.981	122.987	56.267	30.302	175.981	122.987
**12**	**Val**	62.542	32.728	175.915	121.655	62.528	32.741	175.915	121.655
**13**	**His**	56.293	30.995	175.126	123.62	56.332	31.073	175.126	123.62
**14**	**His**	56.46	31.208	175.133	121.81	56.499	31.286	175.133	121.81
**15**	**Gln**	56.035	29.398	175.615	122.2	56.046	29.416	175.615	122.2
**16**	**Lys**	56.321	32.884	176.313	123.133	56.317	32.896	176.313	123.133
**17**	**Leu**	55.229	42.232	177.144	124.695	55.238	42.256	177.144	124.695
**18**	**Val**	62.591	32.587	175.518	121.891	62.577	32.6	175.518	121.891
**19**	**Phe**	57.465	39.537	175.019	123.736	57.441	39.549	175.019	123.736
**20**	**Phe**	57.241	39.658	174.753	122.448	57.217	39.67	174.753	122.448
**21**	**Ala**	52.486	19.103	177.38	125.921	52.475	19.127	177.38	125.921
**22**	**Glu**	56.812	30.349	176.205	120.334	56.817	30.372	176.205	120.334
**23**	**Asp**	54.301	41.108	176.37	121.354	54.315	41.141	176.37	121.354
**24**	**Val**	62.76	32.342	176.958	121.016	62.746	32.355	176.958	121.016
**25**	**Gly**	45.355	**.***	173.709	114.145	45.372	**.***	173.709	114.145
**26**	**Ser**	58.62	63.776	174.615	115.809	58.611	63.798	174.615	115.809
**27**	**Asn**	53.343	38.536	175.316	119.712	53.357	38.562	175.316	119.712
**28**	**Lys**	56.886	32.886	177.145	121.509	56.882	32.898	177.145	121.509
**29**	**Gly**	45.12	0	173.107	111.372	45.137	**.***	173.107	111.372
**30**	**Ala**	52.423	19.243	177.635	124.081	52.412	19.267	177.635	124.081
**31**	**Ile**	60.905	38.622	176.286	120.784	60.895	38.645	176.286	120.784
**32**	**Ile**	61.255	38.697	176.463	126.207	61.245	38.72	176.463	126.207
**33**	**Gly**	45.25	**.***	173.535	114.388	45.267	**.***	173.535	114.388
**34**	**Leu**	55.198	42.379	177.401	122.453	55.207	42.403	177.401	122.453
**35**	**Met**	55.247	32.621	176.152	121.511	55.268	32.667	176.152	121.511
**36**	**Val**	62.535	32.77	176.565	122.356	62.521	32.783	176.565	122.356
**37**	**Gly**	45.359	**.***	173.889	114.064	45.376	**.***	173.889	114.064
38	**Gly**	45.166	**.***	173.517	110.229	45.183	**.***	173.517	110.229
39	**Val**	62.358	32.746	176.389	120.383	62.344	32.759	176.389	120.383
40	**Val**	62.381	32.788	176.091	124.981	62.367	32.801	176.091	124.981
